# Practical guidance for collecting reproducible time-to-event endpoints in retrospective observational oncology studies

**DOI:** 10.3389/fepid.2026.1958489

**Published:** 2026-10-08

**Authors:** Emily C. Zabor, Nathan A. Pennell, Sujata Patil

**Affiliations:** 1 Department of Quantitative Health Sciences, Cleveland Clinic, Cleveland, OH, United States; 2 Bristol Myers Squibb, Lawrenceville, PA, United States

**Keywords:** cancer endpoints, censored data, Cox regression, epidemiological studies, Kaplan–Meier, retrospective studies, survival analysis, time-to-event endpoints

## Abstract

Time-to-event endpoints such as progression-free survival, overall survival, and recurrence-free survival are among the most commonly reported outcomes in cancer research, yet the practical decisions required to collect and structure these data appropriately in retrospective observational studies remain poorly described in the literature. While substantial regulatory and methodological guidance exists for prospective clinical trials, investigators working with data collated retrospectively must navigate consequential decisions about index date selection, event definition, censoring, and follow-up time without equivalent support. Decisions at the data extraction stage can bias results and undermine reproducibility regardless of the rigor of the statistical analysis that follows. In this paper, we provide a practical, decision-focused guide to collating and updating time-to-event endpoints in retrospective oncology studies. Specific topics include how to define the index date, event date, and follow-up end date for different endpoints; what counts as an event and how competing events should be handled; how to manage missing and interval-censored data; how to update outcomes over time; and how to identify potential data quality issues through exploratory analysis and sensitivity testing. We illustrate key concepts using a publicly available lung cancer dataset and a worked example that follows endpoint creation from source records to analysis-ready variables, and we provide a practical checklist that researchers can apply directly to their own work. Our goal is to help epidemiologists, clinicians, and statisticians build time-to-event analyses on a foundation of rigorously collected data, resulting in more reliable and reproducible inference.

## Introduction

Retrospective observational studies are important and common in oncology research. When prospective clinical trials are not feasible, due to cost, rarity of disease, or the need for long follow-up, clinicians and researchers rely on data collated from medical records, registries, and institutional databases to understand disease trajectories and treatment outcomes. Time-to-event endpoints such as progression-free survival, overall survival, and recurrence-free survival are central to these studies, and their analysis commonly requires statistical methods that can handle censored observations (1). These methods include the Kaplan–Meier estimator, Cox regression, and competing risks regression to name a few (2–4).

The statistical methods used to *analyze* survival data are well-documented and widely taught, yet the practical decisions required to *collect and structure* that data appropriately are far less discussed. For prospective oncology clinical trials, regulatory guidance from bodies such as the FDA provides detailed instruction on endpoint definition and data collection (5). Retrospective observational studies, however, are not guided by this framework. Investigators must make consequential decisions, often without formal guidance, about how to define the start of follow-up, what qualifies as an event, how to handle patients whose exact event dates are unknown, and how to update outcome data over time. These decisions are rarely described in detail in published papers, making it difficult for readers to evaluate study quality or for researchers to replicate findings in new datasets (6).

This paper is designed to provide a practical, decision-focused guide to the upstream data extraction choices that can ultimately make a time-to-event analysis robust, and to serve as a complement to existing survival analysis tutorials (7–9). We address the following: how to define and select an appropriate index date, last follow-up date, and event date; what counts as an event and how competing events should be handled; how to update survival data over time; and how to identify potential data quality issues through exploratory analysis and sensitivity testing. Analytic methods enter this discussion only insofar as they determine what must be collected. The choice of analysis often dictates which variables and dates are required, so we reference specific analytic methods to explain data extraction decisions rather than to recommend or fully describe those methods themselves. Throughout, we use a concrete dataset from the North Central Cancer Treatment Group to illustrate key concepts. The Appendix includes a worked example in a small synthetic dataset. This worked example illustrates data extraction decisions used to form an analysis ready dataset. Our aim is to help oncology researchers produce analyses and inferences that are not only statistically sound but built on a foundation of rigorously collected data. Table 1 summarizes the key decision points discussed throughout this paper and may serve as a practical reference for researchers undertaking similar analyses.

**Table 1:** Considerations when collating time-to-event outcomes.

Consideration	Best Practice
1. Index date	• Apply a single, uniform index date definition to all patients,noting that this may differ across endpoints within the same study
	• Verify the index date is clinically meaningful and that for single-event endpoints considered here, all patients are truly event-free at time zero (e.g., date of diagnosis, surgery, or end of treatment)
2. Event definition	• Clearly define what constitutes an event for each endpoint; use published consensus criteria where available (e.g., STEEP for breast cancer)
	• Identify competing events and determine whether a competing risks analysis is warranted
	• Document in advance how ambiguous events will be classified (e.g., death without confirmed recurrence, unknown cause of death)
3. Follow-up end date	• Define the follow-up end date separately for each endpoint — “last known alive” is not equivalent to “last known recurrence-free”
	• Ensure the follow-up end date reflects genuine knowledge of event-free status; using a later date inflates follow-up time and biases results
4. Censoring	• Record the reason for the end of follow-up where it is known, for example administrative cutoff, death, or loss to follow-up, so that whether censoring is likely to be informative can be assessed at the analysis stage.
	• For imaging-detected events, collect both interval start and end dates in case interval censoring methods are needed
5. Missing dates	• For partially known dates, record the level of granularity actually available, for example month or year only, rather than silently entering a full date, so that the handling of partial dates can be pre-specified and varied in sensitivity analyses.
6. Updating outcomes	• When updating, identify all patients who require new data — not just those known to be alive; patients who previously progressed may have new events or longer follow-up
7. Exploratory analysis	• Examine Kaplan–Meier plots with number-at-risk tables, censoring tick marks, and confidence bands
	• Look for crossing curves, large drops at regular intervals, early plateaus, and differential censoring patterns between groups
	• Consider truncating the *x*-axis where fewer than roughly five patients remain at risk in any group, because survival estimates become unstable when few patients remain at risk.
8. Sensitivity analyses	• Pre-specify sensitivity analyses for all key assumptions: alternate censoring dates, alternate event definitions, competing risks approaches, and clinically relevant subgroup analyses

## What is a time-to-event endpoint?

In oncology studies, a frequently analyzed time-to-event endpoint is one where a participant enters a study at a defined starting point and is followed forward in time until the event of interest occurs or observation stops. When the event has not occurred by the time observation stops, the follow-up time is right-censored, a feature of survival data we return to below. Right-censored data is the focus of this guidance. Unlike binary outcomes (did the event occur?) or continuous outcomes (how large is the tumor?), time-to-event endpoints capture both *whether* an event occurred and *when* it occurred, making them particularly informative for understanding disease trajectories and treatment effects over time. In cancer research, commonly evaluated time-to-event endpoints include overall survival (OS), progression-free survival (PFS), recurrence-free survival (RFS), disease-free survival (DFS), and cancer-specific survival (CSS), each defined by a different event type and often by a different index date.

What makes time-to-event endpoints particularly challenging, especially in retrospective observational studies, is that the event of interest is often not observed for every participant. Some patients will still be alive, or free of recurrence, at the time the data are finalized. Others may be lost to follow-up before the study ends. In both cases, we know only that the event had not yet occurred as of the last date the patient was observed, and we do not know whether or when it will occur in the future. These participants are said to be *right-censored* and survival analysis methods such as the Kaplan–Meier estimator and Cox proportional hazards regression are designed to incorporate these censored observations rather than discard them as missing data. Observed data for a time-to-event endpoint consist of two basic elements: the date a participant enters the study (the index date) and either the date the event was observed (the event date) or, if the participant had not yet experienced the event of interest, the date at which follow-up ended (the censor date). The time elapsed between the index date and either the event date or censor date is the participant's follow-up time.

Figure 1 illustrates these concepts using six hypothetical patients with different clinical trajectories. Patient A remains event-free through the end of the study and is censored at the administrative end date. Patient B is lost to follow-up before the study ends and is censored at the last date they were known to be event-free. Patients C and D both die during the study period, contributing an event at their respective dates of death. Patient F experiences disease recurrence during follow-up, contributing a recurrence event at t_F. Patient E also experiences recurrence at t_E and subsequently dies at t_C (the same time point at which Patient C dies). This trajectory illustrates that a single patient may be censored for one endpoint but contribute an event for another. A patient who dies without a documented recurrence, such as Patient C, would be censored for recurrence-free survival but would contribute a death event for overall survival. The appropriate timepoint at which to censor is a question that is discussed in detail later in this paper.

**Figure 1:**
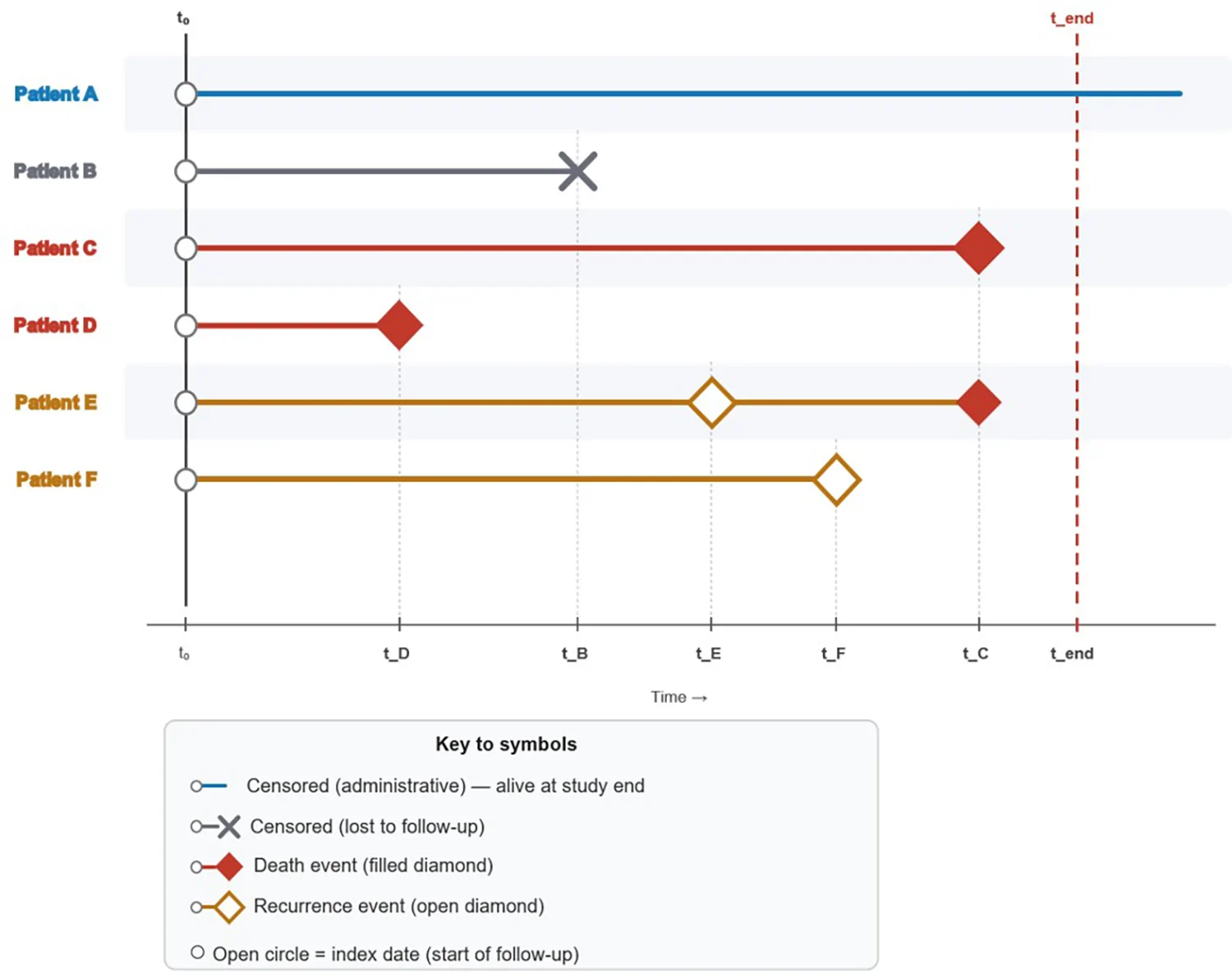
Depiction of time course for six hypothetical study subjects. Depiction of the follow-up trajectory for six hypothetical study subjects. Each patient enters the study at time zero (t₀, denoted by an open circle) and is followed forward until either the event of interest occurs or follow-up ends. The vertical dashed red line marks the study end date (t_end). Patient A remains event-free through the end of the study and is administratively censored at t_end (arrow). Patient B is lost to follow-up before the study ends and is censored at t_B (×). Patients C and D die during the study period, contributing death events at t_C and t_D respectively (solid diamonds). Patient F experiences disease recurrence at t_F (hollow diamond). Patient E experiences recurrence at t_E (hollow diamond) and subsequently dies at t_C. Patient C illustrates death without recurrence and would therefore be censored for a recurrence-free survival endpoint while contributing a death event for overall survival.

In prospective clinical trials, the infrastructure required to capture these elements efficiently and accurately is built into the study design. Visit schedules are standardized, outcome assessments are pre-specified, and data collection is monitored in real time. In retrospective observational studies, these elements must instead be reconstructed from existing records collated from medical charts, institutional databases, cancer registries, or some combination thereof. The decisions made during that reconstruction process, about where follow-up begins, what qualifies as an event, and when a patient should be considered censored, have a direct and consequential impact on the validity of the resulting analysis. These decisions should be made and documented *before* data extraction begins, not resolved on a case-by-case basis during extraction, to avoid the introduction of inadvertent bias. The remainder of this paper addresses each of these decisions.

## Collecting the data

In a perfect scenario, there would be no loss to follow-up, everyone would be observed for the same length of time, and the date of every event would be known with certainty. For most retrospective datasets this is unrealistic. Patients move, change providers, or die outside the healthcare system. Records are incomplete. Scans are missed or performed off-schedule. The practical task of collecting time-to-event data is therefore one of making pre-specified decisions to address the complexity and missingness of such datasets. In this section, we discuss how to define and collect the three data elements required for every time-to-event analysis: the index date, the last follow-up date, and the event date and type. Considerations on how to update time-to-event endpoints are also discussed.

### What is the index date, last follow-up date, and event date?

One of the most important aspects in any time-to-event analysis is identifying the index, the last follow-up date, and the event, which are used to calculate each patient's follow-up time (Figure 1, start date denoted by t_0_). Getting these dates right is important because errors in index date or follow-up end date can directly translate into biased survival estimates and potentially misleading conclusions.

The index date must be defined consistently and applied uniformly to all patients for a given endpoint, though it may differ across endpoints evaluated within the same study. Francis et al. (10) provide a useful illustration in their study of patients with retinoblastoma receiving intra-arterial chemotherapy, in which recurrence-free survival, treatment-free survival, and progression-free survival were all evaluated. The index date for progression-free survival was the date of first visit, while the index date for both recurrence-free survival and treatment-free survival was the end date of chemotherapy. The end date of chemotherapy was a timepoint at which all patients were verifiably free of both disease and treatment. This example illustrates how the index date must be a point in time at which all patients are genuinely at risk for the event of interest and have not yet experienced it. Using an index date at which some patients may have already experienced the event introduces *immortal time bias*. For example, this bias can exist when group membership (i.e., response to treatment) is defined by something that can only be ascertained after the index date (i.e., treatment start) so that the event-free period required to *reach* that classification is wrongly credited to the group. One way to avoid this is a landmark analysis, in which follow-up is restarted at a fixed post-index time point and only patients still event-free at that landmark are included. Other approaches, such as treating the classifying variable as time-dependent or time-dependent matching, may be preferable depending on the question (11). The relevant point for data extraction is that the time at which the post-baseline characteristic is ascertained must be recorded, since every one of these analytic approaches requires it (12). There are no universal rules governing the correct index date; it will depend on the disease, the treatment course, and the research question. What is essential is that the chosen date is clinically meaningful, uniformly applied, and explicitly documented in the methods.

Once the index date has been established, the follow-up end date and event date must be determined for each endpoint separately. A patient is censored when the event of interest has not occurred by the end of the observation period and no further information is available. As illustrated in Figure 1, censoring can arise for different reasons. Patient A is censored at the administrative end of the study, while Patient B is censored earlier due to loss to follow-up. These two types of censoring have different implications for a key assumption regarding censoring that underlies the Kaplan–Meier estimator and Cox regression. The Kaplan–Meier estimator assumes that censoring is independent of the event time. Cox regression relaxes this to conditional independence, requiring only that censoring be independent of the event time given the covariates in the model. In both methods the concern is that the reason a patient is censored may itself be related to their risk of the event, thereby violating the censoring assumption. Administrative censoring, where follow-up ends because the study closes, generally satisfies this assumption. Loss to follow-up is more problematic since if patients are lost because they became too ill to attend clinic, or because they sought care elsewhere after experiencing a recurrence, then censoring could be informative and standard methods may produce biased estimates. Whenever possible, the reasons for loss to follow-up should be collected, examined and reported.

It is important to be aware that follow-up end dates may differ across endpoints. Oppong et al. (13) provide a useful example in their study of metformin use and outcomes in diabetic women with breast cancer, in which both recurrence-free survival and overall survival were evaluated. The event date for recurrence-free survival was the earlier of the recurrence date or the death date, while the event date for overall survival was the date of death. Correspondingly, the follow-up end date for recurrence-free survival was the last date the patient was known to be recurrence-free, while the follow-up end date for overall survival was the last date the patient was known to be alive. A common mistake is to use “last known alive” as the follow-up end date for recurrence-free survival as well. Unless a scan was performed and confirmed negative on that date, the patient was known to be alive but not genuinely known to be recurrence-free. Assigning a follow-up end date beyond the point at which a patient is truly known to be event-free artificially extends their event-free time and produces overly optimistic survival estimates. This is a subtle but important form of bias that is easy to introduce and difficult to detect after the fact.

A related complication is *interval censoring*, which arises when the exact date of an event cannot be determined and can only be localized to an interval between two observation time points. This is particularly common for imaging-detected endpoints such as disease progression and recurrence, where the last negative scan date and the first positive scan date bracket the true event date but do not identify it precisely. Lycke et al. (14) encountered this in their study of cervical cancer risk in women with cervical intraepithelial neoplasia grade 2. Cervical cancer can develop without symptoms and in this study, the onset of disease was defined as occurring somewhere in the interval between the last disease-free biopsy and the date of cancer diagnosis. Standard survival methods assume that event times are known exactly and treat the scan date at which the event was detected as the event date. This is an approximation that is usually reasonable when scan intervals are short and consistent, but that can introduce meaningful bias when intervals are long or vary systematically by treatment group or other covariates. Specialized methods for interval-censored data exist and are described in detail elsewhere (15); the practical recommendation here is simply to collect both the start and end dates of the relevant interval during data extraction, so that the option to apply these methods remains available if the statistician determines they are warranted at the time of analysis.

Finally, a practical issue that arises frequently in retrospective data is partial date information. These are situations in which only the month or year of an event is available rather than the full date. When extracting data, it is important is to record the available granularity rather than silently substituting a full date, because that record is what allows the ambiguity to be handled transparently later. How partial dates are then resolved is an analytic decision. Midpoint assignment (e.g., the 15th of the month if day is missing) is one common approach, but it is not always the best or a conservative choice, and the appropriate handling depends on the analysis. Whatever approach is chosen should be pre-specified, and sensitivity analyses using alternative assignments and analyses with and without the affected patients should be reported so that conclusions do not rest on the assumption.

### What counts as an event in a time-to-event analysis?

Defining what constitutes an event is as important as defining when follow-up begins, and the two decisions are closely intertwined. For overall survival the answer is unambiguous since death from any cause is an event, and patients still alive at last follow-up are censored. For endpoints that incorporate disease recurrence or progression the definition becomes considerably more complex. Should the event be based on clinical assessment alone, radiologic confirmation, or both? How should a patient who dies with signs of recurrence but without a formal assessment having been completed be classified? What if imaging findings are equivocal? These questions do not have universal answers, but they must be answered explicitly and consistently before data abstraction begins. Leaving them unresolved and making case-by-case judgments during data extraction introduces subjectivity that can bias results, reduce reproducibility, and make it difficult for readers to evaluate or replicate the analysis.

Where published consensus definitions exist, they should be used. In breast cancer, the Standardized Definitions for Efficacy End Points (STEEP) criteria, first published in the Journal of Clinical Oncology in 2007 and updated in 2021, provide a detailed and expert-validated accounting of what constitutes an event for each endpoint of interest (16, 17). The criteria were updated to reflect new treatments and scientific insights. Using established criteria of this kind enables comparability with the broader published literature. Investigators working in disease settings where no equivalent consensus criteria exist should document their event definitions with comparable rigor and consider seeking input from disease-specific experts before finalizing them. The event definition should be reported in sufficient detail in the methods section such that another investigator could apply the same criteria to a different dataset.

An additional consideration is the potential presence of *competing events*. A competing event is any event that precludes the occurrence of the primary event of interest (18). Consider a study of advanced lung cancer where the primary endpoint is cancer-specific mortality, and death from other causes (cardiovascular disease, treatment toxicity) is a competing event. These are mutually exclusive because once a patient dies from another cause, cancer-specific death cannot occur. Standard Kaplan–Meier analysis censors these deaths as uninformative, which overestimates cancer-specific mortality. This is a well-identified issue in allogeneic hematopoietic stem cell transplantation studies, and in older or heavily comorbid populations (19, 20). When competing events are non-negligible, the cumulative incidence function (CIF) and Fine-Gray subdistribution hazard regression are among the alternatives to the Kaplan–Meier estimator and Cox regression (21). They are not the only options, and the appropriate choice depends on the research question. The relevant point for data collection is that if competing events may be analyzed, the type and date of each competing event must be captured during abstraction so that these methods remain available to the analyst.

Once event criteria have been established, a plan for handling missing event data should be developed before data extraction begins. A common scenario in oncology is cancer-specific survival, where only deaths attributable to the cancer under study are counted as events. In such analyses it is not unusual to have a subset of patients whose death is confirmed but whose cause of death is unknown. Excluding these patients entirely risks introducing bias if they differ systematically from those with known cause of death. Censoring them or assigning them to an “unknown” category in a competing risks framework is a common method but has been shown to induce bias (22, 23). The best approach is to design data abstraction to minimize missing cause-of-death information from the outset. In practice this means recording, for every death, the date of death, the cause as documented, the source of that cause information, and a flag for whether the cause is confirmed, presumed, or unknown. Capturing the source and confirmation status, rather than a single cause field, is what allows the analyst to distinguish a well-documented cause from an assumed one and to run the sensitivity analyses described below. Where feasible, abstraction can also supplement chart review with death certificate data or linkage to national mortality registries, which can resolve causes that chart abstraction alone leaves unknown. When missing data remain, pre-specified sensitivity analyses are essential. At minimum, analyses should be conducted assuming all patients with unknown cause of death died from the cancer of interest, assuming none did, and under one or more intermediate scenarios. The degree to which results vary across these scenarios and among important subgroups provides a transparent and quantitative basis for assessing confidence in the primary findings.

### Updating survival data

In oncology research it is common to periodically update clinical outcomes by returning to medical records or other data sources to capture events and follow-up time that have accumulated since the original data extraction. Updating outcomes increases the number of observed events, extends follow-up time, and improves statistical power, all of which strengthen the reliability of the analysis. The process of updating, however, requires careful planning that is specific to the endpoints of interest and that should follow the same pre-specified decision rules applied during the original data extraction. If overall survival is the only endpoint, updating is relatively straightforward. Records are reviewed for all patients not yet known to have died, and any deaths occurring during the additional follow-up period are recorded. When both progression-free survival and overall survival are of interest, the process is more comprehensive. All patients except those already known to have died without prior progression require review. Among patients who had previously progressed, some may have died during the additional follow-up period, generating new overall survival events; others may have had additional clinical contact, extending their follow-up time for overall survival. Patients who were censored for both endpoints at the time of original data extraction may have experienced new progression or death events or may simply have a later censoring date.

A practical recommendation is to document the updating process explicitly and record the date on which the update was conducted, the data sources reviewed, and the decision rules applied to any ambiguous cases. This documentation supports reproducibility, facilitates future updates, and provides an audit trail that can be valuable during peer review or if questions arise after publication. For statisticians, it is also worth noting that a data update effectively changes the analysis dataset and triggers a full re-run of all pre-specified analyses, including sensitivity analyses.

## Exploratory and sensitivity analyses

With the data collected and updated, focus shifts to exploratory analyses that can reveal potential data quality issues and to sensitivity analyses that assess the robustness of findings. This important task is a shared responsibility of the clinician and the statistician. The clinician brings the disease-specific knowledge needed to recognize when a pattern is clinically implausible, while the statistician brings the methodological tools to diagnose and address what the data reveal.

### Identifying possible data issues

One of the first and most informative steps in any time-to-event analysis is a careful visual examination of the Kaplan–Meier survival curves. This should be done for the overall study population and, where relevant, separately for any subgroups intended for comparison. At this exploratory stage, the plot should always include three elements that are sometimes omitted in published papers due to publication requirements but are extremely important. These elements include the number-at-risk table below the *x*-axis, censoring tick marks along the survival curves, and pointwise confidence bands. Each of these conveys distinct information, as described below.

#### Number-at-risk table

The number-at-risk table shows how many patients remain under observation at each time point and is essential for understanding how confident we can be in the estimates at any given time. As follow-up progresses and patients experience events or are censored, the number at risk decreases, and survival estimates based on very small numbers are unstable and potentially misleading. A practical rule of thumb is to truncate the *x*-axis at the point beyond which fewer than five patients remain at risk in any group, though this threshold may be adjusted upward in smaller studies. In the example dataset, only one female and two males remained at risk at 30 months, suggesting the *x*-axis should be cropped at 24 months (Figure 2).

**Figure 2:**
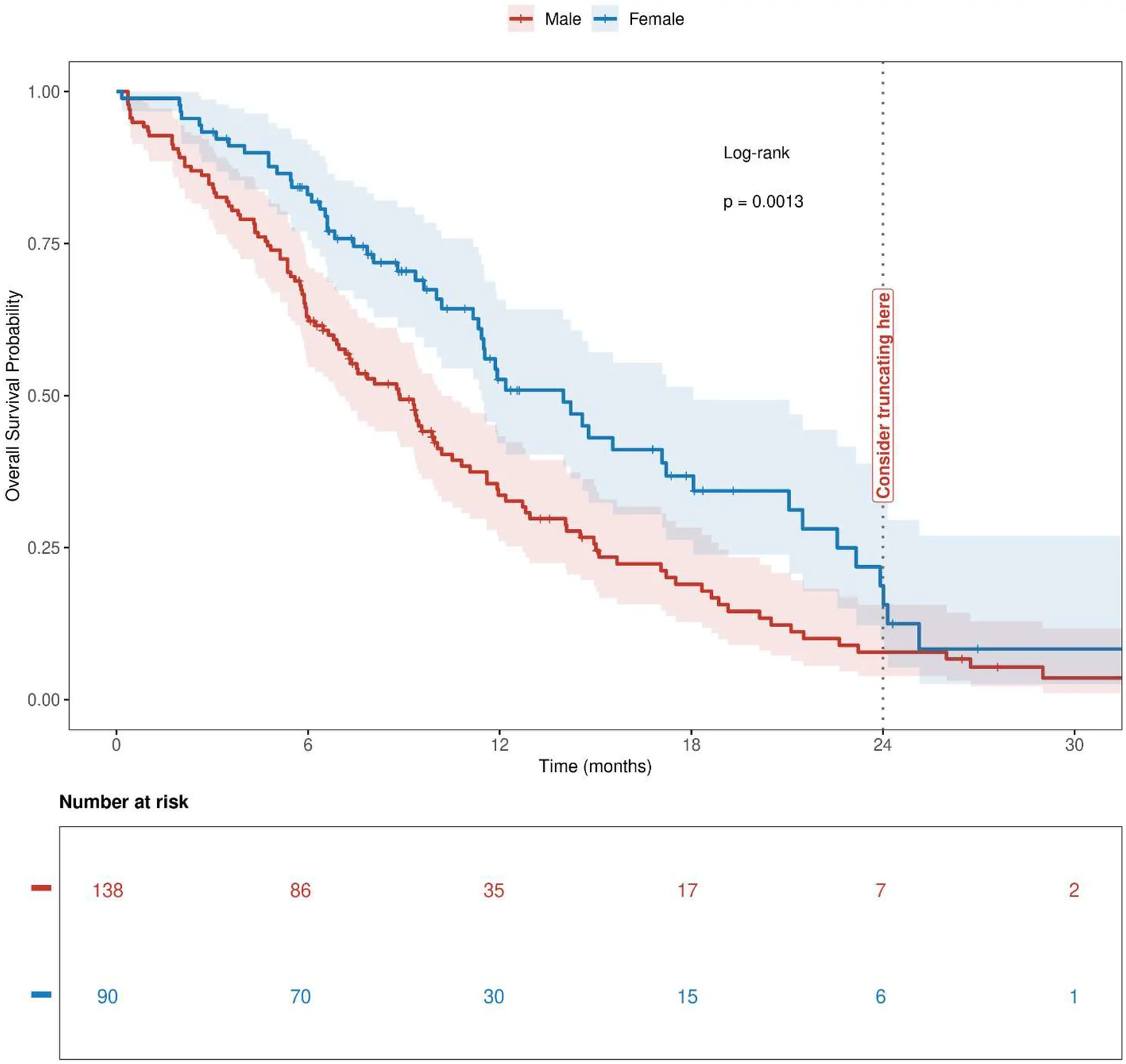
Example of Kaplan-Meier plot with details such as censoring tick marks, confidence intervals, numbers of events and at risk added, for diagnosing potential data issues. Kaplan–Meier estimates of overall survival by sex in the example lung cancer dataset (North Central Cancer Treatment Group, *n* = 228), with male patients shown in red and female patients in blue. The shaded regions represent 95% pointwise confidence bands. Censoring times are indicated by tick marks along each curve. The number-at-risk table below the plot shows the number of patients remaining under observation at each time point for each group. The *x*-axis is displayed through 30 months; at this point only one female and two male patients remain at risk, suggesting truncation at 24 months should be considered for final reporting (dotted vertical line). The log-rank *p*-value for the comparison between sexes is shown. Median overall survival was 14.0 months in females and 8.9 months in males, corresponding to a hazard ratio of 0.59 (95% CI: 0.42–0.82) with male patients as the reference group, indicating a lower instantaneous hazard of death in female patients.

#### Censoring tick marks

The pattern of censoring tick marks along the survival curves reveals when and how frequently patients are being censored, and whether this pattern differs across groups. Differential censoring is where patients in one group are censored at a systematically higher rate or at systematically different time points than patients in another group. This type of censoring is a potential source of bias and can undermine the non-informative censoring assumption on which standard survival methods rely. If differential censoring is observed, its potential causes and implications should be discussed explicitly. Large clusters of censoring tick marks at specific time points can also reveal administrative features of the data, such as a data lock date or a change in follow-up frequency, that are worth understanding before proceeding to formal analysis.

#### Confidence bands

Pointwise confidence bands convey the uncertainty in the survival estimates at each time point and widen as the number at risk decreases. Examining the confidence bands helps identify time periods during which estimates are too imprecise to support meaningful inference, which can also inform where to truncate the plot for display.

Beyond these three elements, the shape of the survival curves themselves carries important information. Figure 3 points out a few of the issues that can be diagnosed from the Kaplan–Meier graph. Large, abrupt drops in the curve at regular time intervals can suggest that events are being detected at scheduled assessment visits rather than continuously and is a form of interval censoring discussed earlier in this paper (24). Extended plateaus in the curve, particularly at the beginning of follow-up, may indicate a mismatch between the index date and the earliest time at which events can realistically be observed, or may suggest that a covariate of interest is time-dependent in a way that requires a landmark analysis or a time-dependent modeling approach can address (12, 25). Plateaus that appear later in follow-up may reflect genuine long-term survivors in the population or may reflect an administrative data cutoff beyond which events are no longer being recorded.

**Figure 3:**
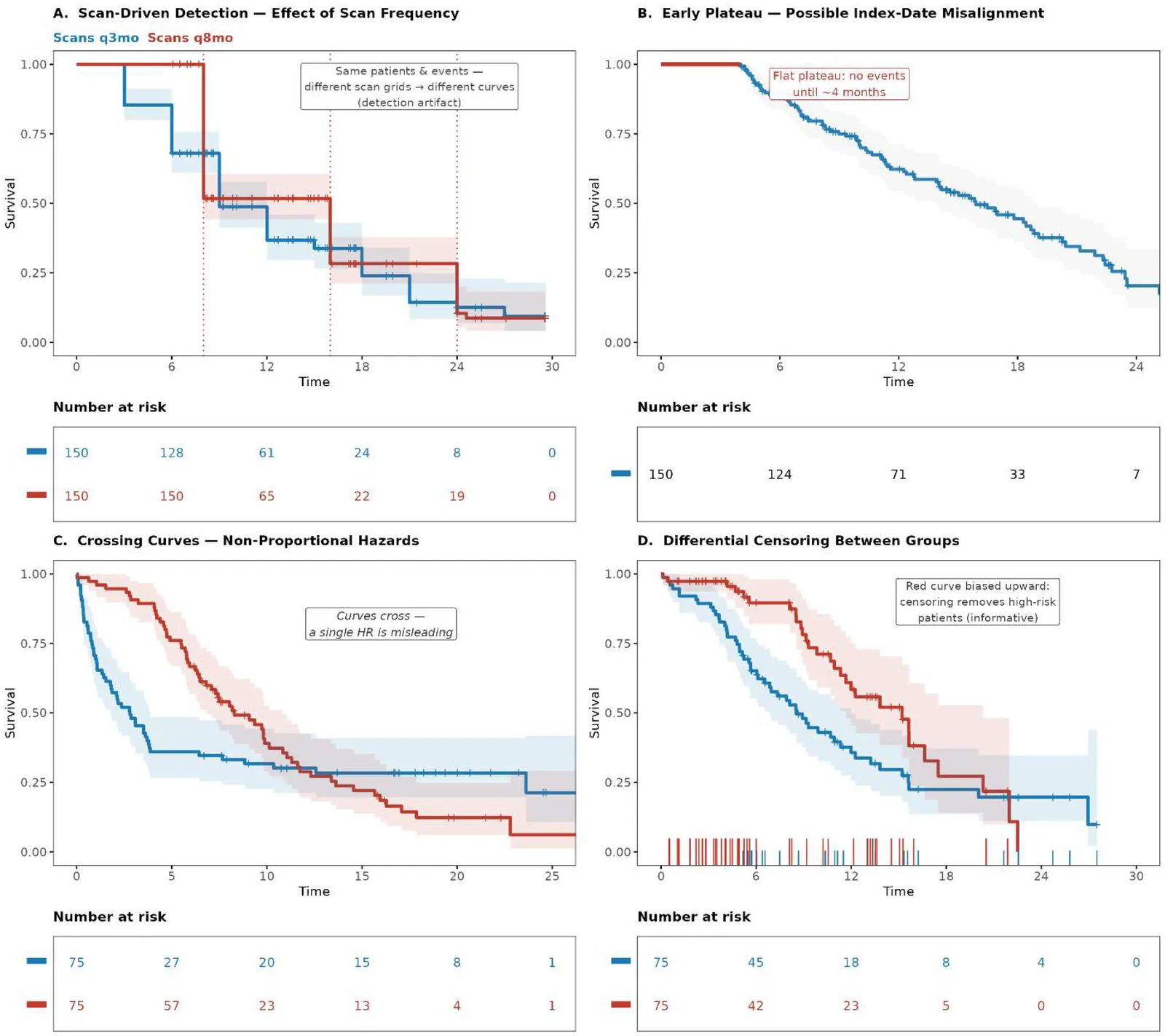
Visual diagnostics for KM Curves. Illustrative Kaplan–Meier curve patterns that can indicate potential data quality issues. Panel **(A)** Large, abrupt drops in the survival curve at regular six-month intervals suggest that events are being detected at scheduled imaging assessments rather than continuously. This is a form of interval censoring that can introduce bias. Panel **(B)** An extended plateau at the beginning of follow-up, with no events recorded in the first three months, may indicate a mismatch between the index date and the earliest time at which events can realistically be observed, or the presence of a time-dependent covariate requiring a landmark analysis approach. Panel **(C)** Crossing survival curves indicate that the direction of the association between the grouping variable and outcome reverses over time, suggesting non-proportional hazards and a single summary hazard ratio may be misleading in this setting. Panel **(D)** Differential patterns in censoring tick marks between groups (both with similar hazards) can indicate that patients in one group are being censored at a systematically higher rate or at different time points, which may violate the non-informative censoring assumption underlying standard survival analyses.

When comparing two or more groups on the same plot, the relative behavior of the curves over time is informative. Curves that cross suggest that any single summary hazard ratio may be misleading, as the direction of the association between the grouping variable and outcome reverses over time. Curves that diverge early and remain separated suggest a consistent treatment or group effect throughout follow-up. Curves that are initially separated but converge later may indicate an early effect that diminishes over time.

### Sensitivity analyses

Sensitivity analyses are a fundamental component of rigorous inference. Their purpose is to assess how dependent the primary results are on the specific decisions and assumptions made during data extraction and analysis, and to provide readers with a transparent basis for evaluating the robustness of the conclusions. We describe these analyses here not to define how the final analysis should be conducted, but because each one can only be performed if the data it requires were captured during collection. The appropriate sensitivity analyses will depend on the specific context of each study, but the following categories are some that can be applied to retrospective oncology analyses. The data extraction implications are noted in each category.

#### Alternate index and censoring dates

Because the index date and follow-up end date are often judgment calls, analyses using plausible alternative date definitions can reveal how sensitive results are to these choices. Large changes in estimated hazard ratios or survival probabilities across alternate date specifications should prompt reflection on which definition best reflects clinical reality. For this to be possible, the candidate index and follow-up end dates must be captured during collection, not only the single date chosen for the primary analysis.

#### Alternate event definitions

Where the event definition involves ambiguous cases, sensitivity analyses using more inclusive and more restrictive event definitions provide a range of plausible results and quantify the uncertainty introduced by these ambiguities. This requires that the components of the event, such as the recurrence date and the death date, be recorded separately rather than collapsed into a single event flag.

#### Competing risks

If a meaningful proportion of patients experience a competing event, a sensitivity analysis applying competing risks methods should be conducted alongside the primary analysis. If results are demonstrably different, both analysis methods might require inclusion in a manuscript. The data collection implication is that the type and date of each competing event must be captured, not only the primary event.

#### Missing data

As discussed earlier, pre-specified sensitivity analyses for missing event data are essential when the missing attribution frequency is non-trivial. These analyses are possible if missing values are collated, that is, whether a value is missing is coded as such, since these details allow the analyst to run an analysis under alternative scenarios.

#### Landmark analyses

When a covariate of interest is only defined or meaningful after the index date, for example response to treatment, which can only be assessed after treatment is administered, immortal time bias arises if that time is credited to the exposed group. A landmark analysis is one way to address this, restarting follow-up at a pre-specified post-index time point and including only patients still event-free at that point. Other approaches exist, and the choice depends on the question. For data collection the implication is the same regardless of method, the ascertainment time of the post-index characteristic must be captured (12).

#### Subgroup analyses

When subgroup inference is of interest, the variables that deine those subgroups must be captured at collection with the same rigor as the primary variables. Any such analyses should be pre-specified where possible and interpreted with appropriate caution given the reduced sample sizes and increased risk of false positive findings.

Across all of these, the goal is not to find a specification that produces the most favorable result, but to understand the range of results that are consistent with the data under plausible assumptions. Large variation in results across sensitivity analyses should not be considered a failure to find a conclusion, but rather, these analyses provide important information about the limits of what we can infer from the data.

## Conclusion

Time-to-event analyses are among the most powerful and widely used tools in oncology research, but their validity depends critically on decisions made long before any statistical model is fit. In retrospective observational studies, where data are reconstructed from existing records rather than prospectively collected under controlled conditions, these decisions about the index date, event definitions, censoring rules, and follow-up end dates can be critical. Reporting guidelines for observational research, notably the STROBE statement (26), specify *what* should be reported but do not provide guidelines involved in data abstraction decisions that can have an impact on statistical inference. Errors at the data collating stage can be complex and introduce bias that may not be able to be corrected in the analysis phase.

Several limitations of this guidance should be noted. Our focus is the construction of right-censored, single-event endpoints from existing records, and we do not address recurrent-event endpoints, in which an individual may experience the event more than once. The collating and analysis of these types of endpoints raise important considerations beyond the scope of this paper. We likewise do not treat left-truncated or multistate data, which require their own design and analytic handling. Our illustrations also draw on a single publicly available dataset, so some situations that arise in specific disease settings or data sources will not be represented. Finally, the guidance here concerns which information to collect and how to structure it, not which analytic method to apply. The appropriate analysis depends on the research question, the target estimand, and the data at hand, and those choices should be made with a statistician rather than read off a checklist.

In summary this paper provides oncology researchers and statisticians with a practical, decision-focused guide to collecting, updating, and interpreting right-censored time-to-event endpoints. Throughout, the importance of pre-specifying all data collection decisions before extraction begins, documenting those decisions explicitly, and conducting sensitivity analyses that characterize the uncertainty introduced by unavoidable assumptions is emphasized. The checklist provided in Table 1 and the keyword definitions in Table 2 distill these recommendations into a format that can be applied directly to any retrospective oncology study involving time-to-event endpoints. However, it is important to keep in mind that adherence to any checklist does not substitute for careful, context-specific thinking about the unique features of each dataset and research question. The checklist is intended as a starting point for planning, not a replacement for methodological judgment. Rigorous data abstraction following the guidance provided here will result in analyses that are more reproducible, more interpretable, and more likely to contribute meaningfully to the evidence base that guides patient care.

**Table 2:** Glossary of key terms, definitions, and common pitfalls in time-to-event analysis for retrospective oncology studies.

Term	Definition	Common pitfall
Index date	The starting point of follow-up, from which time-to-event is measured	Using different index date definitions for different patients within the same endpoint; using an index date at which some patients have already experienced the event
Event date	The date on which the event of interest was observed	Using the date of clinical suspicion rather than confirmed assessment; inconsistent event confirmation criteria across patients
Censor date	The last date on which a patient was known to be event-free, for patients who did not experience the event	Using “last known alive” as the censor date for endpoints other than overall survival (e.g., using it as the recurrence-free censor date without scan confirmation)
Follow-up time	The time elapsed between the index date and either the event date or censor date	Calculating follow-up time using all patients regardless of event status, which conflates follow-up time with time-to-event
Right censoring	Censoring that occurs when a patient's follow-up ends before the event is observed, and the event may occur at some unknown future time	Excluding censored patients from the analysis, which in the settings encountered in practice biases the estimated survival, typically downward, because longer event times are disproportionately censored.
Interval censoring	Censoring that occurs when the event is known to have occurred between two observation time points but the exact date is unknown	Using the date of event detection (e.g., scan date) as the exact event date without acknowledging the interval, particularly when scan intervals are long or vary by group
Left truncation	Can occur when individuals become part of the risk set only after a delay, so that individuals who experienced the event before that entry time are never observed.	Treating delayed entry times as if follow-up began at time zero, or assuming that affected individuals must be dropped. Risk-set adjustment can accommodate left truncation under appropriate assumptions.
Immortal time bias	Bias introduced when time during which a patient could not have experienced the event (e.g., time before treatment initiation) is incorrectly attributed to the treated group	Assigning follow-up time before treatment initiation to the treated group, artificially inflating their survival time
Non-informative censoring	The assumption that the reason for censoring is unrelated to the patient's subsequent risk of the event	Ignoring informative censoring (e.g., patients dropping out because they became too ill), which violates a key Cox model assumption
Proportional hazards	The assumption that the hazard ratio between groups remains constant over time	Fitting a Cox model without checking the proportional hazards assumption; interpreting a single hazard ratio when curves cross or converge
Hazard ratio (HR)	The ratio of the instantaneous event rate between two groups at any point in time, among patients still at risk	Interpreting the hazard ratio as a risk ratio or odds ratio; reporting a hazard ratio without checking proportionality
Competing event	Any event that precludes the occurrence of the primary event of interest	Treating competing events (e.g., death) as censored observations in a Kaplan–Meier analysis, which overestimates cumulative incidence of the primary event
Median follow-up	The median observation time among patients who did not experience the event	Calculating median follow-up across all patients regardless of event status, which conflates follow-up time with time-to-event
Median survival	The time at which the survival probability equals 50%	Reading median survival from raw data rather than from the Kaplan–Meier curve; reporting it without a confidence interval
X-time survival	The probability of remaining event-free beyond a specified time point, estimated from the Kaplan–Meier curve	Choosing time points *post hoc* based on where estimates look favorable; reading values from raw data rather than the survival curve
Events per variable (EPV)	The ratio of observed events to predictor variables in a multivariable regression model	Including too many predictors relative to the number of events, leading to overfitting and unstable estimates
Reverse Kaplan–Meier	A method for estimating median follow-up time by swapping the event and censoring indicators	Using median follow-up among all patients rather than event-free patients or reverse Kaplan–Meier, producing a misleading summary
Landmark analysis	An analysis in which follow-up begins at a pre-specified time point after the index date, restricted to patients who have reached that time point event-free	Analyzing a time-dependent covariate (e.g., treatment response) as if it were measured at baseline, introducing immortal time bias
Sensitivity analysis	An analysis conducted under alternative assumptions to assess the robustness of the primary findings	Conducting sensitivity analyses *post hoc* and selectively reporting only those that confirm the primary result

## Data Availability

Publicly available datasets were analyzed in this study. This data can be found here: https://stat.ethz.ch/R-manual/R-devel/library/survival/html/lung.html.
