# Supplementary material for: Practical guidance for collecting reproducible time-to-event endpoints in retrospective observational oncology studies
Source: Front Epidemiol. 2026 Oct 8;6:1958489. doi: 10.3389/fepid.2026.1958489 (PMC13687036; doi:10.3389/fepid.2026.1958489)
Supplement: Supplementary file 1 [file Table1.docx]

**Appendix. A worked example going from source records to an analysis ready dataset**

The main text describes the data extraction decisions required to build and analyze a time-to-event endpoint. This appendix works through those decisions on a small set of synthetic patient records to show how each one is made, documented, and translated into the final dataset. The patient records are fictitious and are constructed to contain the kinds of incompleteness and conflicting information that arise in practice. The example concerns a hypothetical retrospective observational study of patients with resected non-small cell lung cancer. Two endpoints are of main interest, recurrence-free survival and overall survival. Cancer-specific survival is an endpoint that will be analyzed as well. Outcome data are abstracted from the electronic health record. Surveillance imaging was performed roughly every six months. The administrative data lock date is December 31, 2022. The decision rules below were fixed before abstraction began and map directly onto the checklist in Table 1.

**Pre-specified decision rules**

1. Index date. The date of surgical resection is time zero for both endpoints, because that is the point at which every patient is rendered disease-free and first becomes at risk for recurrence.
2. Recurrence-free survival event. Recurrence must be confirmed by unequivocal imaging or by pathology. Imaging described only as concerning or equivocal, without confirmation, does not count as an event. Death from any cause is treated as an event for the primary recurrence-free survival analysis, so the endpoint is a composite of recurrence or death.
3. Overall survival event. Death from any cause is the event.
4. Censoring. For recurrence-free survival, a patient with no event is censored at the date of last disease assessment, meaning the last imaging or clinical evaluation that established recurrence-free status. For overall survival, a patient with no event is censored at the date last known alive.
5. Partial dates. When only month and year are recorded, the fifteenth of the month is used as a working date, and the first and last day of the month are carried as sensitivity bounds.
6. Interval capture. For imaging-detected recurrence, both the last negative assessment date and the first positive assessment date are recorded, so that interval-censoring methods remain available.
7. Cause of death. For any analysis that treats non-cancer death as a competing event, cause of death is classified as cancer, non-cancer, or unknown. Cause is taken from the death certificate where available and from the treating oncologist's final note otherwise. Cause is not inferred from the absence of documentation.

**Source records**

The abstracted records for seven patients are shown below.

| **Patient** | **Resection** | **Surveillance imaging and findings** | **Other contact** | **Death** |
| --- | --- | --- | --- | --- |
| A | Feb 12, 2019 | Neg Aug 2019, neg Feb 2020, positive recurrence Sep 3, 2020 |  | Died Jun 15, 2021, lung cancer |
| B | Mar 5, 2019 | Neg Sep 2019, oncology note records recurrence on imaging from 6/2020 | Clinic Nov 10, 2022, alive |  |
| C | Apr 20, 2019 | Neg through last scan Oct 10, 2020, no further surveillance | Clinic Aug 15, 2022 for COPD, alive, no imaging |  |
| D | Jun 1, 2019 | Neg through last scan Dec 5, 2020 |  | Died Mar 10, 2021, myocardial infarction, no recurrence documented |
| E | February 2019 (day not recorded) | Radiology Jan 2021 concerning for recurrence, biopsy Feb 2021 benign, neg scan Aug 20, 2021, then no further records |  |  |
| F | May 15, 2019 | Neg Nov 2019, neg May 20, 2020, Nov 2020 scan missed, positive recurrence Aug 5, 2021, then no further records |  |  |
| G | Jul 8, 2019 | Neg Jan 2020, neg Jul 2020, neg Feb 2021, then no further surveillance |  | Died Nov 2021, cause not documented, death recorded in outside records |

**Walking through the decisions**

Patient A is the clean case. Recurrence is confirmed on imaging, so recurrence-free survival has an event at the September 2020 scan. Death follows and provides the overall survival event. No ambiguity arises.

Patient B carries a partial recurrence date. The note localizes recurrence to June 2020 without a day. The midpoint rule assigns June 15, 2020 as the working recurrence date, with June 1 and June 30 held as sensitivity bounds. The patient is alive at data lock, so overall survival is censored at the last date known alive, the November 2022 clinic visit.

Patient C is the case the main text warns about most directly. The last disease assessment is the negative scan in October 2020. The patient was later seen in clinic in August 2022 for an unrelated respiratory problem and was alive, but no imaging was performed. Under the pre-specified rule, recurrence-free survival is censored at the last disease assessment in October 2020, not at the later clinic visit. Overall survival is censored at the August 2022 visit, because that visit establishes the patient was alive even though it does not establish recurrence status. The two endpoints therefore have different censoring dates for the same patient.

Patient D died of myocardial infarction with no documented recurrence. Under the primary composite definition, this death is a recurrence-free survival event at the March 2021 date. Overall survival has an event at the same date. How this patient is handled changes if the endpoint is defined differently, which we return to below.

Patient E shows conflicting records. Imaging in January 2021 was read as concerning for recurrence, but the biopsy in February 2021 was benign. The pre-specified rule requires confirmation, so this is not a recurrence event and the patient remains at risk. A later scan in August 2021 was negative and is the last disease assessment. The resection date is recorded only to the month, so the midpoint rule places index at February 15, 2019.

Patient F illustrates why interval width matters. As with any imaging-detected recurrence, the true recurrence time is not usually observed directly. It lies somewhere between the last negative scan and the first positive scan, so the recurrence is interval-censored. For Patients A and B that interval is roughly the routine surveillance gap of about six months, and using the detection date as the event date is a reasonable approximation. For Patient F the November 2020 scan was missed, so the interval runs from the last negative scan in May 2020 to the first positive scan in August 2021, a span of about fourteen months. Using the August 2021 detection date as a point event now discards a large and irregular window of uncertainty. Recording both the last negative and first positive dates, as the interval-capture rule requires, preserves the information an analyst would need to apply interval-censoring methods if the wide interval exists is an issue throughout the dataset.

Patient G shows a death with unknown cause. Surveillance was negative through February 2021, and the patient died in November 2021, but the cause is not recorded in the available notes and the death is known only from an outside record. Under the composite recurrence-free survival definition this death is an event, so the primary recurrence-free survival analysis is unaffected by the missing cause. The cause matters only for the alternative definition that treats non-cancer death as a competing event. Under the pre-specified rule the cause is classified as unknown rather than assumed, and the patient is carried into the sensitivity analyses described below. The day of death is also missing, so the midpoint rule places it at November 15, 2021, with month bounds retained.

**Analysis-ready dataset**

Applying the rules yields the following. Times are in months from the resection date. The documentation column records the decision so the dataset carries its own audit trail.

| **Patient** | **Index** | **RFS time** | **RFS event** | **OS time** | **OS event** | **Cause of death** | **Documentation** |
| --- | --- | --- | --- | --- | --- | --- | --- |
| A | Feb 12, 2019 | 18.7 | 1 | 28.1 | 1 | Cancer | Recurrence confirmed on imaging |
| B | Mar 5, 2019 | 15.4 | 1 | 44.2 | 0 | Not applicable, alive | Partial recurrence date, midpoint applied, bounds 14.9 and 15.9 |
| C | Apr 20, 2019 | 17.7 | 0 | 39.9 | 0 | Not applicable, alive | RFS censored at last disease assessment, OS at last known alive |
| D | Jun 1, 2019 | 21.3 | 1 | 21.3 | 1 | Non-cancer | Death without recurrence, event under composite definition |
| E | Feb 15, 2019 | 30.1 | 0 | 30.1 | 0 | Not applicable, alive | Concerning scan not confirmed by biopsy, not counted as event |
| F | May 15, 2019 | 26.7 | 1 | 26.7 | 0 | Not applicable, alive | Recurrence interval 12.2 to 26.7 recorded |
| G | Jul 8, 2019 | 28.3 | 1 | 28.3 | 1 | Unknown | Death, cause unknown, recorded as unknown rather than assumed |

This is a small toy data example. It records the captured fields required for main analyses, including the cause of death that the competing-risks and unknown-cause sensitivity analyses depend on. It does not add columns that could be used to explore different types of analyses. Knowing the analysis plan apriori means working closely with the statistician to ensure data collated are sufficient for analysis.

**Updating the data**

Suppose the study is refreshed two years later, with a new data lock date of December 31, 2024, and the update is conducted in early 2025. Updating follows the same pre-specified rules used at the original abstraction. The confirmation requirement for recurrence, the censoring definitions, and the partial-date rule are all reapplied unchanged, so that the updated records are constructed on the same basis as the originals.

The first task is to identify who requires review. Patients A, D, and G were already known to have died at the original lock, so both of their endpoints are complete and no review is needed. The remaining four patients, B, C, E, and F, are reviewed. This illustrates the point made in the main text that updating is not limited to patients known to be alive and event-free. Updating also requires looking at patients who already had an event for one endpoint but remain at risk for another.

Patient B had already contributed a recurrence-free survival event at the original data lock, so that endpoint is complete. Review of the interval since the last contact finds that the patient died of lung cancer on April 18, 2023. This is a new overall survival event. The overall survival record changes from censored at 44.2 months to an event at 49.4 months. The recurrence-free survival record is unchanged.

Patient C had no event for either endpoint at the original lock. Review finds that surveillance imaging resumed, with a negative scan in March 2023 and a confirmed recurrence on September 12, 2023. Because this recurrence meets the same confirmation rule applied originally, it is a new recurrence-free survival event, and the record changes from censored at 17.7 months to an event at 52.8 months. Following the interval-capture rule, the last negative date and first positive date are recorded, giving a recurrence interval of 46.8 to 52.8 months. The patient remains alive, last known alive at a clinic visit in November 2024, so the overall survival record extends from 39.9 to 66.6 months and remains censored. Patient C is the most instructive case in the update, because a patient who contributed no information to the recurrence-free survival analysis at the original lock now contributes a confirmed event.

Patient E illustrates the opposite outcome. Review finds no new records of any kind. The patient has no further imaging, no clinical contact, and no death record. The follow-up time does not change simply because calendar time has passed. Patient E remains censored at 30.1 months for both endpoints, exactly as before. Updating extends follow-up only where new information exists, and assigning a later censoring date in the absence of new contact would repeat the last-known-alive error described earlier.

Patient F had already contributed a recurrence-free survival event, so that endpoint is complete. Review finds additional clinical contact, with the patient alive at a visit in May 2024 and no death recorded. The overall survival record extends from 26.7 to 60.5 months and remains censored.

The updated dataset is summarized below, with changes noted in the last column.

| **Patient** | **RFS time** | **RFS event** | **OS time** | **OS event** | **Cause of death** | **Change at update** |
| --- | --- | --- | --- | --- | --- | --- |
| A | 18.7 | 1 | 28.1 | 1 | Cancer | None, already complete |
| B | 15.4 | 1 | 49.4 | 1 | Cancer | New OS event, was censored at 44.2 |
| C | 52.8 | 1 | 66.6 | 0 | Not applicable, alive | New RFS event, was censored at 17.7. OS extended from 39.9 |
| D | 21.3 | 1 | 21.3 | 1 | Non-cancer | None, already complete |
| E | 30.1 | 0 | 30.1 | 0 | Not applicable, alive | None, no new records found |
| F | 26.7 | 1 | 60.5 | 0 | Not applicable, alive | OS extended from 26.7, remains censored |
| G | 28.3 | 1 | 28.3 | 1 | Unknown | None, already complete |

As recommended in the main text, the update is documented with the date it was conducted (the new data lock), the sources reviewed, and the rules applied to any ambiguous records. Because the update changes the analysis dataset, adding two events and extending follow-up for two patients, it triggers a full re-run of all pre-specified analyses, including any sensitivity analyses.

**How different decisions can change results**

The dataset above reflects one set of decisions. Reasonable alternatives produce different numbers, which is why the choices must be pre-specified and documented.

Consider the index date. If the date of diagnosis rather than resection were used, every follow-up time would lengthen by the interval between diagnosis and surgery, typically several weeks. More importantly, that pre-surgical interval is time during which recurrence of a resected tumor is not yet defined, because the patient has not been rendered disease-free. Using diagnosis as the index date for recurrence-free survival would credit patients with event-free time before they were at risk, which is the mechanism of immortal time bias. The resection date avoids this.

Consider Patient C under a naive censoring rule. If recurrence-free survival were censored at the last known alive date rather than the last disease assessment, this patient would contribute 39.9 months of event-free follow-up instead of 17.7. That is more than 22 months of follow-up during which the patient was known to be alive but was not known to be recurrence-free. In a full cohort, repeating this error across many patients inflates the recurrence-free survival estimate.

Consider Patient D under a different event definition. If recurrence-free survival were defined as recurrence only, with death from other causes as a competing event rather than an event, this patient would no longer contribute a recurrence-free survival event. In a naive Kaplan-Meier analysis the patient would instead be censored at the death date, which overestimates the cumulative incidence of recurrence. A competing-risks analysis would treat the death as a competing event. In this small illustration the choice moves the number of recurrence-free survival events, and in a real cohort with a meaningful fraction of non-cancer deaths the effect on the estimated curves can be substantial.

The same alternative definition raises a second question for Patient G. If non-cancer death is treated as a competing event, the analysis must decide how to handle a death whose cause is unknown. Assigning it to cancer death, to non-cancer death, or to a separate unknown category each produces a different cumulative incidence estimate, and the three can diverge materially when unknown-cause deaths are common. This is why the cause is recorded as unknown at collection rather than resolved by assumption. The recorded value is what allows the analyst to run the pre-specified sensitivity analyses. If the abstraction coded the death as non-cancer on the basis of the myocardial-infarction pattern seen in Patient D, sensitivity analyses would not have been possible.

**How exploratory analysis sends you back to the source**

Two simple checks on the assembled dataset reveal problems that require returning to the records.

The first is a comparison of each patient's recurrence-free survival censoring date against the date of last disease assessment. For most patients these coincide. For Patient C the naive rule would have produced a censoring date far beyond the last assessment. Flagging that gap prompts a return to the source of the data to confirm that no interim imaging exists before accepting the long follow-up time. In this case none exists, and the shorter time is what is used for analysis.

The second is a summary of recurrence detection dates against the surveillance schedule. Patient F's gap between the last negative and first positive scan stands out. That gap can prompt a return to the source of the data to check whether imaging was performed elsewhere during the missed interval, since an outside scan could narrow the interval and change the recorded event date.
